# The Non-Destructive Test of Steel Corrosion in Reinforced Concrete Bridges Using a Micro-Magnetic Sensor

**DOI:** 10.3390/s16091439

**Published:** 2016-09-06

**Authors:** Hong Zhang, Leng Liao, Ruiqiang Zhao, Jianting Zhou, Mao Yang, Runchuan Xia

**Affiliations:** 1 College of Civil Engineering, Chongqing Jiaotong University, Chongqing 400074, China; superww058@163.com (H.Z.); Runchuan727@163.com (R.X.); 2 School of Materials Science and Engineering, Chongqing Jiaotong University, Chongqing 400074, China; lengliao@cqjtu.edu.cn (L.L.); rqzhao@cqjtu.edu.cn (R.Z.); 3 College of Civil Engineering, Chongqing Three Gorges University, Chongqing 404100, China; yangmao199104@163.com

**Keywords:** steel corrosion, non-destructive test, micro-magnetic sensor, self-magnetic flux leakage, numerical simulation

## Abstract

This paper presents a non-destructive test method for steel corrosion in reinforced concrete bridges by using a 3-dimensional digital micro-magnetic sensor to detect and analyze the self-magnetic field leakage from corroded reinforced concrete. The setup of the magnetic scanning device and the measurement mode of the micro-magnetic sensor are introduced. The numerical analysis model is also built based on the linear magnetic charge theory. Compared to the self-magnetic field leakage data obtained from magnetic sensor-based measurement and numerical calculation, it is shown that the curves of tangential magnetic field at different lift-off height all intersect near the edge of the steel corrosion zone. The result indicates that the intersection of magnetic field curves can be used to detect and evaluate the range of the inner steel corrosion in engineering structures. The findings of this work propose a new and effective non-destructive test method for steel corrosion, and therefore enlarge the application of the micro-magnetic sensor.

## 1. Introduction

Reinforced concrete is the most popular construction material in the world and widely used to bridge construction due to the advantage of strong bearing, low-cost, and easy construction. However, a major issue for reinforced concrete structures is the corrosion of the reinforcement steel bars exposed to aggressive environmental conditions, such as a humid, saline-alkaline climate. The steel corrosion makes a significant contribution to the failure of an engineering structure. Approximately 40% of damages of engineering structure result from the steel corrosion and there is a loss of ~14 billion dollars per year just in the United States. Therefore, the evaluation of corrosion in reinforced concrete is very important for the management and maintenance of engineering structures. The traditional nondestructive test techniques, such as electrochemical method [1], linear ultrasonic testing (UT) [2], eddy current testing (ECT) [3], infrared thermography (IRT) [4], and X-ray diffraction (XRD) [5], are all time-consuming technology or need expensive equipment for determining corrosion in the depth of reinforced concrete.

Reinforcing steel bars are made from a typical ferromagnetic material, which is a very important component of the reinforcing concrete structure. A defect (e.g., cracking, fatigue failure, stress concentration and corrosion) in ferromagnetic materials can change the structure of magnetic domains and their macro-properties are consequently changed, such as magnetoconductivity, coercive force, and hysteresis. This effect can be used for different applications in material science, especially in the field of nondestructive testing by using magnetic measurement to evaluate the status of the steel material in engineering structures, in particular to detect the stress-concentration, corrosion, and failure-originated zones. A number of nondestructive magnetic techniques have been developed, such as magnetic Barkhausen emission (MBE) [6], magnetoacoustic emission (MAE) [7], stress-induced magnetic anisotropy (SMA) [8], and magnetic field leakage (MFL) [9,10,11,12,13]. The physical mechanics of these techniques requires a strong magnetic field to magnetize the specimen for testing, and then detect induced-related phenomena to evaluate the health status of a specimen, so these techniques could be called active magnetic test methods. On the other hand, the shape and density of stimulated magnetic fields differ from the characteristic of tested objects. These techniques are time-consuming and even difficult to operate for some irregular structures [14]. For meeting the requirement of developing a more simple and effective magnetic technique in engineering, a positive magnetic method called metal magnetic memory (MMM) technique was proposed by A. A. Doubov in 1997 [15]. The advantage of MMM technique is that the Earth’s magnetic field instead of an artificial strong field is used as the stimulus source. Under the effect of the earth field and mechanical loads/defects, the self-magnetic field leakage (SMFL) signals are generated from corroded or stress-concentration regions where the tangential component reaches a maximum and the normal component transfers its polarity and has a zero value. The MMM technique is suitable for many engineering practices [16,17,18], but up to now has only been used as a qualitative test technique to determine the possible dangerous positions without quantitative results. The more accurate and quantitative criteria are deficient and required for the MMM technique.

In the present work, we introduce a passive magnetic test method to detect and evaluate the corrosion of reinforcement steel bars in the reinforced concrete structure by detecting the SMFL from the corrosion region. When a local corrosion happens, it brings a strong local corrosion pressure for expanding of corrosion products, which breaks the magnetic continuity and improves the magnetic resistivity of corrosion parts for the material loss and the magnetic-stress coupling [16,19]. The magnetic permeability is accordingly changed because of the stress-magnetic effect of iron as a ferromagnetic material. As a consequence, the SMFL is generated from the corrosion zone. By detecting and analyzing the SMFL signal produced by the corrosion, the position and status of corrosion in the reinforced concrete structure can be determined. This method does not need the equipment to actively excite a magnetic field, it is an effective, time-saving, and easy-operation method to non-destructively test the corrosion in the reinforced concrete structure.

The paper is organized as follows. In Section 2, the experimental setup and theoretical model based on the micro-magnetic sensor are introduced. The experimental results and calculated data of the SMFL signals are shown in Section 3, and the quantitative relationship between leaked magnetic field and steel corrosion is given. In Section 4, we summarize all these results.

## 2. Experimental Setup and Theoretical Model Based on Micro-Magnetic Sensor

### 2.1. Experiment Details Based on Micro-Magnetic Sensors

**Figure 1:**
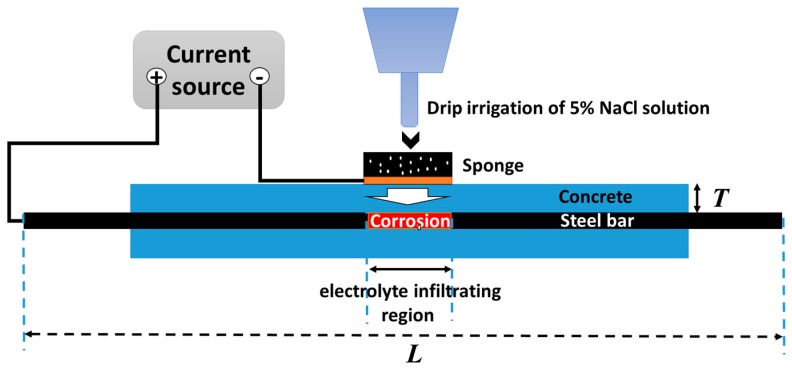
The schematic of electrochemical corrosion.

The corroded specimens of reinforced concrete are prepared by electrochemical method as shown in Figure 1. A steel bar is enwrapped by concrete, whose thickness T = 3 cm and the steel bar length L = 150 cm. The corrosion level can be controlled by the corroded current and time according to the Faraday law and the corrosion length can be controlled by the size of region infiltrated by electrolyte (5% NaCl solution).

**Figure 2:**
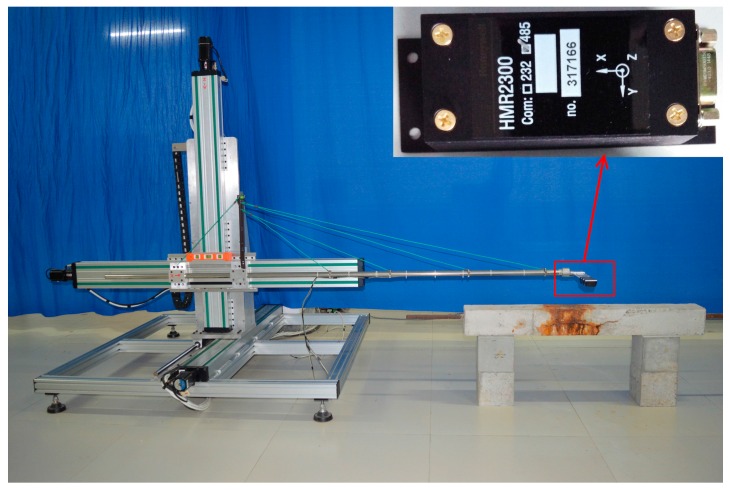
The 3D scanning device for magnetic field measurement based on the micro-magnetic sensor. The inset shows the model of the micro-magnetic sensor.

Figure 2 shows the self-designed 3-dimensional (3D) scanning device for magnetic field measurement based on the 3D mechanical displacement system and the high-precision micro-magnetic sensor. The mechanical displacement system consists of 3D aluminum track and bracket system, stepping motor driver, and a hollow bar for supporting the sensor. The displacement accuracy of the stepping motor driver is as high as 0.1 mm. To improve the stability of the supporting bar when moving, a cable-stayed-like structure was used in the scanning device. The Honeywell HMR 2300 magnetometer is employed as the micro-magnetic sensor. It is a three-axis smart digital magnetometer, and the three axes oriented in orthogonal directions of HMR 2300 can measure the X, Y and Z vector components of a magnetic field. The output range of this micro-magnetic sensor is ±2 Gs with a resolution to less than 70 μGs.

**Figure 3:**
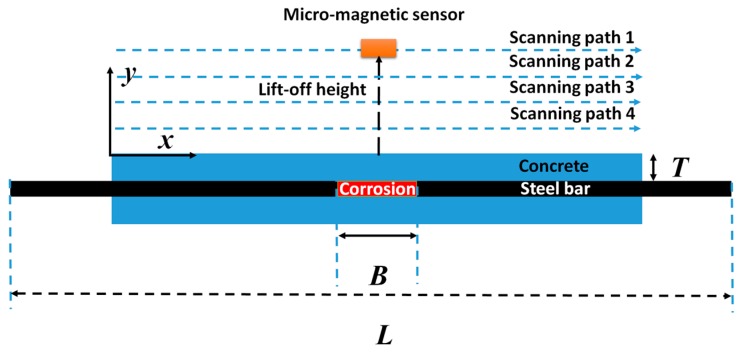
The location and scanning paths of the micro-magnetic sensor.

Two reinforced concrete samples are corroded under a current intensity of 0.5 A and the corrosion time is 96 h, 120 h, and 144 h, respectively; while the corrosion length is ~20 cm and ~15 cm for the sample #1 and #2, respectively. The location and scanning paths of magnetic sensor imbedding on the 3D scanning device are shown in Figure 3, where the *H*_x_ component of magnetic field is defined as a tangential field. Figure 3 shows four scanning paths at different lift-off height (LFH) to scan the magnetic field around the reinforced concrete sample.

### 2.2. Theoretical Model for Measured Data

In order to establish a physical model for the SMFL signals from a corrosion region, a linear model considering the redistribution of magnetic charge in the corrosion zone is presented. Magnetic charge is an equivalent model, which has been developed to simulate the magnetic field leakage [20,21,22,23]. For a ferromagnet, its exterior magnetic field would be considered to originate from the magnetic charge: ρ=−∇·M. ***M*** is the magnetization satisfying $M=\left(\mu_{r}-1\right)H_{\mathrm{mL}}$, where *μ*_r_ is the relative magnetic permeability and ***H***_mL_ is the Weiss field which is the effective field producing self-magnetization in the ferromagnet. It is obvious that the quantity of charge is decided by the magnetization ***M***, and then is only decided by *μ*_r_ when the Weiss field ***H***_mL_ is fixed. In other words, the magnetic charge is changed with the magnetic permeability of the material. For the steel bar under a local mechanical stress, the magnetic permeability would change due to the stress-magnetic effect. As a result, magnetic charge changes accordingly in these zones. For simplicity, here a phenomenological model is built in which a linear charge distribution is adopted.

**Figure 4:**
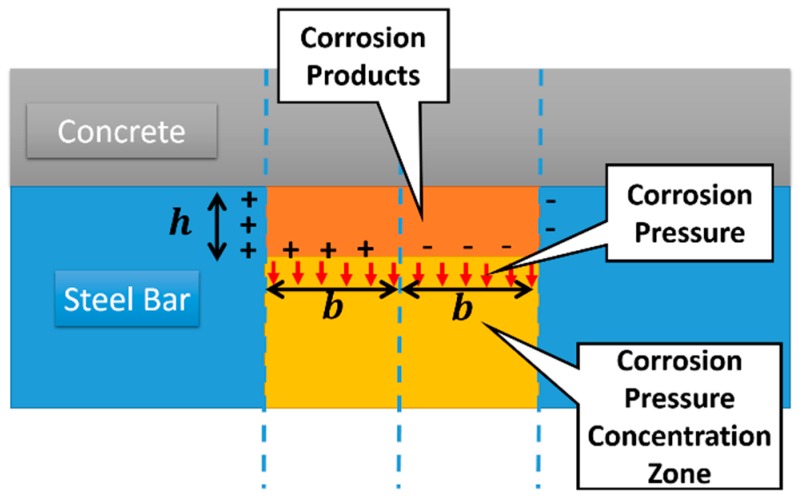
The schematic of the magnetic charge distribution and corrosion pressure at the corrosion region on locally corroded steel bar enwrapped by concrete.

As shown in Figure 4, we assume a defect with width 2*b* and depth *h* at the bar surface when the steel bar is partially lost due to the corrosion. Under the earth magnetic field, the dislocated magnetic charge is concentrated at both ends of the corrosion zone. Moreover, the corrosion induces a strong pressure stress on the steel bar and there exists a local stress concentration in the corrosion zone. For this reason, a redistribution of magnetic charge occurs at the bottom of the corrosion zone. The magnetic charge density in the corrosion zone can be expressed as

(1) $$\rho (x,y)=\{\begin{array}{c} \rho_{\max},(x=-b,-h<y<0)\\ -\rho_{\max},(x=b,-h<y<0)\\ -\rho_{\max}\frac{x}{b},(-b<x<b,y=-h) \end{array}$$

where *ρ*_max_ denotes the maximum charge density, and the charge density is considered as the uniform distribution at the edges of the corroded region for simplicity.

**Figure 5:**
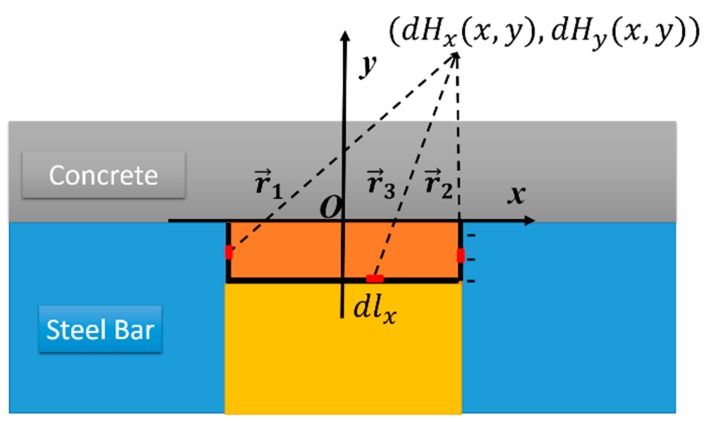
The self-magnetic leakage field generated from the magnetic charge at the corrosion region.

As shown in Figure 5, the self-magnetic leakage field at a space point (*x*, *y*) generated by the charge elements of the three regions can be expressed by Equation (2), respectively.

(2) $$\begin{array}{l} dH_{1}(x,y)=\frac{\rho (x^{\prime },y^{\prime })dy^{\prime }}{2\pi \mu_{0}{r_{1}}^{2}}r_{1}\\ dH_{2}(x,y)=\frac{-\rho (x^{\prime },y^{\prime })dy^{\prime }}{2\pi \mu_{0}{r_{2}}^{2}}r_{2}\\ dH_{3}(x,y)=\frac{\rho (x^{\prime },y^{\prime })dx^{\prime }}{2\pi \mu_{0}{r_{3}}^{2}}r_{3} \end{array}$$

where *μ*_0_ denotes the magnetic permeability in the air, ***r***_1_, ***r***_2_, and ***r***_3_ denote the space vectors from the charge element to the space point, respectively.

Thus, the *x* and *y* components of total leaked magnetic field at space point (*x*, *y*) can be obtained by the integral of Equation (2). It can be expressed as

(3) $$\begin{array}{ll} H_{x}(x,y)= & \frac{\rho_{\max}}{2\pi \mu_{0}}\left(\arctan(\frac{y+h}{x+b})-\arctan(\frac{y}{x+b})\right)\\ & -\frac{\rho_{\max}}{2\pi \mu_{0}}\left(\arctan\frac{y+h}{x-b}-\arctan\frac{y}{x-b}\right)\\ & +\frac{\rho_{\max}}{2\pi \mu_{0}b}\left(2b-(y+h)\left(\arctan\frac{b-x}{y+h}-\arctan\frac{-b-x}{y+h}\right)\right)\\ & +\frac{\rho_{\max}}{2\pi \mu_{0}b}\left(\frac{x}{2}\ln\frac{{\left(b-x\right)}^{2}+{\left(y+h\right)}^{2}}{{\left(b+x\right)}^{2}+{\left(y+h\right)}^{2}}\right)\\ H_{y}(x,y)= & \frac{\rho_{\max}}{4\pi \mu_{0}}\ln\frac{{\left(x+b\right)}^{2}+{\left(y+h\right)}^{2}}{{\left(x+b\right)}^{2}+y^{2}}\\ & -\frac{\rho_{\max}}{4\pi \mu_{0}}\ln(\frac{{\left(x-b\right)}^{2}+{\left(y+h\right)}^{2}}{{\left(x-b\right)}^{2}+y^{2}})\\ & -\frac{\rho_{\max}}{2\pi \mu_{0}b}\left(\frac{\left(y+h\right)}{2}\ln\frac{{\left(b-x\right)}^{2}+{\left(y+h\right)}^{2}}{{\left(b+x\right)}^{2}+{\left(y+h\right)}^{2}}\right)\\ & -\frac{\rho_{\max}}{2\pi \mu_{0}b}\left(x\left(\arctan\frac{b-x}{y+h}-\arctan\frac{-b-x}{y+h}\right)\right) \end{array}$$

## 3. Results and Discussion for Steel Corrosion Based on Micro-Magnetic Sensor

**Figure 6:**
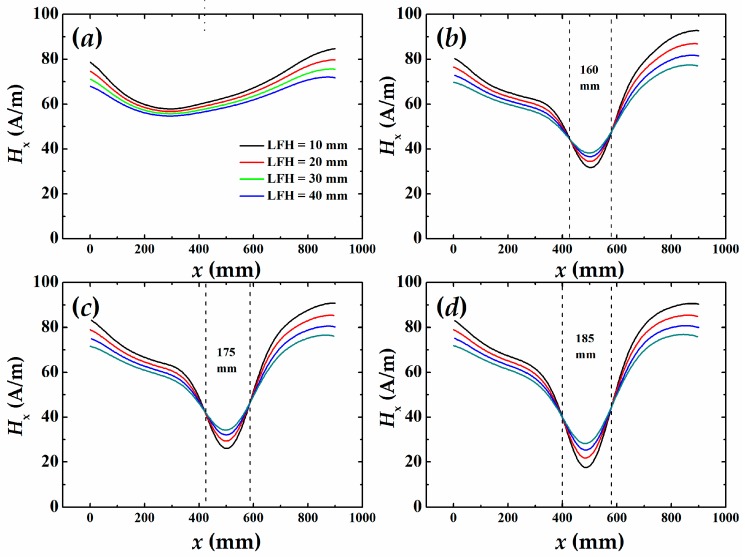
The self-magnetic field around the sample #1 depends on the location of sensor at different lift-off height (LFH): (**a**) non-corroded; (**b**) corroded for 96 h; (**c**) corroded for 120 h; (**d**) corroded for 144 h.

**Figure 7:**
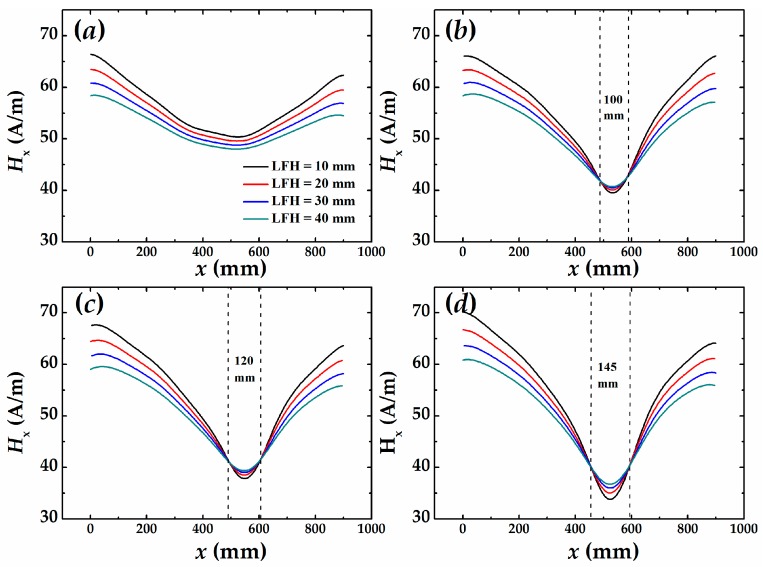
The self-magnetic field around the sample #2 depends on the location of sensor at different LFH: (**a**) non-corroded; (**b**) corroded for 96 h; (**c**) corroded for 120 h; (**d**) corroded for 144 h.

First, we scanned the magnetic field around the two samples and obtained the tangential field (*H*_x_) distributions as shown in Figure 6 and Figure 7, respectively. For the existence of demagnetization field of steel bar and the earth magnetic field, the *H*_x_ is nonzero, but the *x*-*H*_x_ curves at different LFH are almost parallel to each other for no damaged condition. There is no curve intersection, as shown in Figure 6a and Figure 7a, which is a typical *H*_x_ distribution for a healthy steel bar. Figure 6b–d plot the *H*_x_ dependent on the sensors position of sample #1 for the corrosion time of 96 h, 120 h, and 144 h, respectively. It is obvious that all the curves with different LFH intersect to two points when the samples are corroded. The distances between two intersecting points are 16.0 cm, 17.5 cm, and 18.5 cm for the different corrosion time, respectively. The longer the corrosion time is, the closer the distance is to the length of corrosion region. A similar result is found from the measurement for sample #2 for which curves are shown in Figure 7b–d. The result indicates that the position and regions of corrosion can be obtained by analyzing the SMFL signals outside of reinforced concrete.

**Figure 8:**
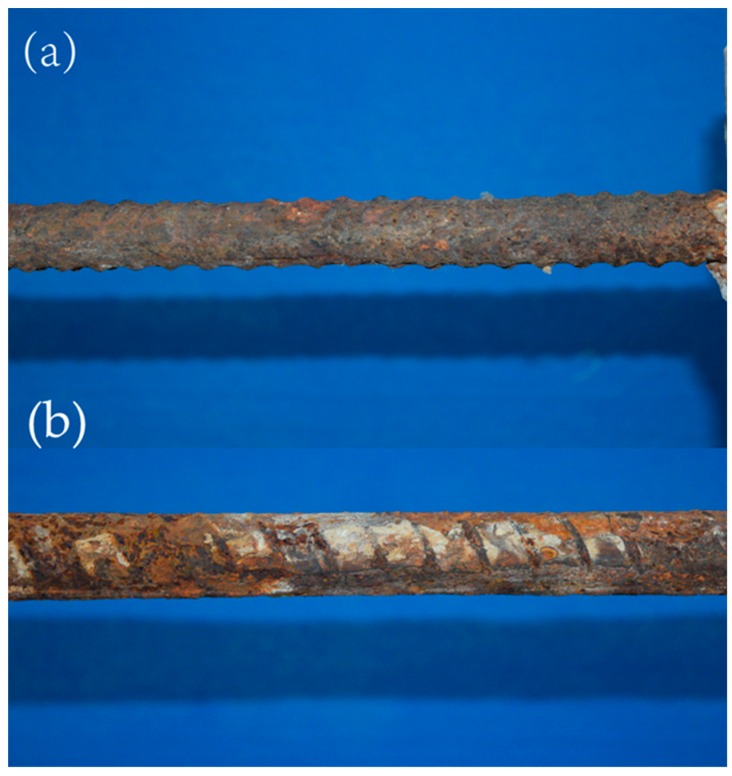
The topography of corroded steel bar after removal of the cover concrete: (**a**) sample #1; (**b**) sample #2.

For testing the real corroded area, the partial concrete has been removed to expose the corroded zone of the steel bar after magnetic detection. The morphology of the corrosion zone is shown in Figure 8. It can be found that the corrosion is actually not serious because the average corrosion depth is ~2 mm. Even the screw threads of the steel bar are not completely removed in the corroded zone. This means that the material removal is not the leading factor for the SMFL, but the corrosion pressure is.

**Figure 9:**
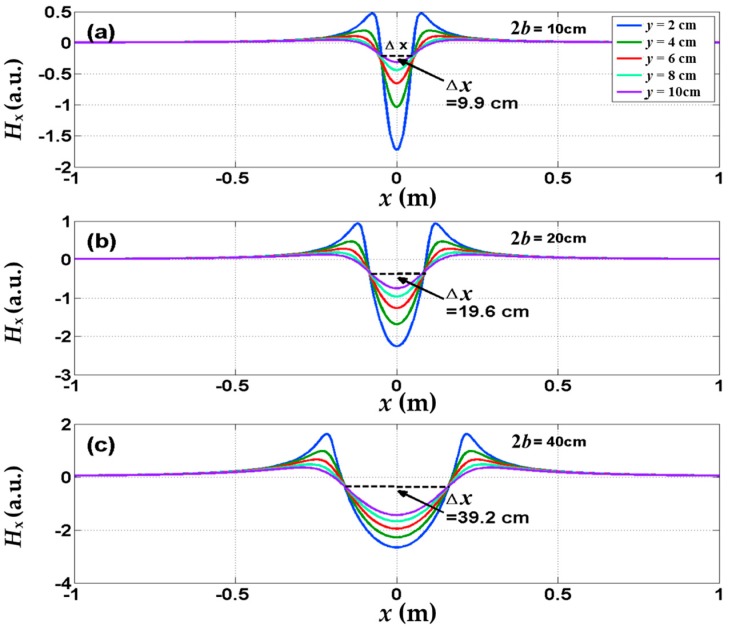
The calculated tangential *H*_x_ of self-magnetic leakage field at the different lift-off height (*y* = 2 cm, 4 cm, 6 cm) for the corrosion length 2*b* = 10 cm, 20 cm, 40 cm.

For better understanding the experimental results, the tangential component *H*_x_ of self-magnetic leakage field outside of concrete can be determined according to Equation (3). Figure 9 shows the *H*_x_ distributions affected by the corrosion length (the value of 2*b* in the Figure 4). Clearly, the phenomena observed in the experiment have been captured in this model. The peaks of *x*-*H*_x_ exhibit the center of the corrosion region. Furthermore, it can be found that the *x*-*H*_x_ curves at different lift-off height (the value of *y* in Figure 5) all intersect at the same two points observed in the experiments. The distance Δ*x* between the intersecting points is enlarged by increasing corrosion length and Δ*x* almost equal to the corrosion length.

## 4. Conclusions

In this work, by detecting and analyzing the SMFL signals outside of corroded reinforced concrete using a micro-magnetic sensor, we find that the *x*-*H*_x_ curves obtained at different lift-off heights all intersect at the same points and the distances between intersecting points are basically equal to the length of the corrosion region. By a linear magnetic charge model, the distribution of *H*_x_ component is simulated and the intersection of curves is also found in the calculated results. This means that the corrosion improves the magnetic resistivity of the steel bar and produces magnetic charge concentration in the corrosion region, which generates this abnormal magnetic field distribution. The results propose a new magnetic NDT technique to detect and evaluate the inner corrosion in engineering structures using high-resolution micro-magnetic sensors. This method has many advantages over traditional techniques. It is a simple, inexpensive and efficient method to non-destructively test the corrosion in the reinforced concrete structure.
